# Pedicle subtraction osteotomy with patient-specific instruments

**DOI:** 10.1016/j.xnsj.2021.100075

**Published:** 2021-08-29

**Authors:** Marco D. Burkhard, Daniel Suter, Bastian Sigrist, Philipp Fuernstahl, Mazda Farshad, José Miguel Spirig

**Affiliations:** a Department of Orthopedics, Balgrist University Hospital, University of Zurich, Switzerland; b Research in Orthopedic Computer Science (ROCS), University Hospital Balgrist, University of Zurich, Zurich, Switzerland; c University Spine Center Zürich, Balgrist University Hospital, University of Zurich, Switzerland

**Keywords:** Spine, Pedicle subtraction osteotomy, Sagittal imbalance, Spinal osteotomy, Patient-specific, 3D-print

## Abstract

**Background:**

Although the utility of patient-specific instruments (PSI) has been well established for complex osteotomies in orthopedic surgery, it is yet to be comparatively analyzed for complex spinal deformity correction, such as pedicle subtraction osteotomy (PSO).

**Methods:**

Six thoracolumbar human cadavers were used to perform nine PSOs using the free-hand (FH) technique and nine with PSI (in total 18 PSOs). Osteotomy planes were planned on the basis of preoperative computed tomography (CT). A closing-wedge angle of 30° was targeted for each PSO. Postoperative CT scans were obtained to measure segmental lordosis correction and the deviation from the planned 30° correction as well as the osseous gap of posterior elements.

**Results:**

The time required to perform a PSO was 18:22 (range 10:22–26:38) min and 14:14 (range 10:13–22:16) min in the PSI and FH groups, respectively (p = 0.489). The PSI group had a significantly higher lordosis gain (29°, range 23–31° vs. 21°, range 13–34°; p = 0.015). The lordosis gain was significantly more accurate with PSI (deviation angle: 1°; range 0–7°) than with the FH technique (9°; range 4–17°; p = 0.003). PSI achieved a significantly smaller residual osseous gap of the posterior elements (5 mm; range 0–9 mm) than the FH group (11 mm; range 3–27 mm; p = 0.043).

With PSI, an angular difference of 3° (range 1–12°), a translational offset of 1 (range 0–6) mm at the level of the lamina, and a vertebral body entry point deviation of 1 (range 0–4) mm was achieved in the osteotomies.

**Conclusions:**

PSI-guided PSO can be a more feasible and accurate approach in achieving a planned lordosis angle than the traditional FH technique in a cadaver model. This approach further reduced osseous gaps, potentially promoting higher fusion rates in vivo.

## Background

Adult spinal deformities with sagittal imbalance have been a major cause of pain and decreased quality of life. Various surgical techniques have been developed to restore lumbar lordosis, with pedicle subtraction osteotomy (PSO) being among those with the highest potential [1]. PSO, which involves a V-shaped closing-wedge osteotomy through the posterior elements, pedicles, and vertebral body, was first described by Eivind Thomasen in 1985 for the correction of lumbar kyphosis in ankylosing spondylitis [2]. Since then, it has been performed in different variations for several conditions, with the fixed sagittal imbalances due to iatrogenic misaligned lumbar fusion being the most common condition [3].

According to Bridwell et al., the osteotomy is closed in such a way that bone-on-bone contact is accomplished in the posterior, middle, and anterior columns [4,5]. In general, a lordosis gain of 25–35° can be expected following a PSO [1,4]. The procedure aims to not gain as much lordosis as possible but achieve an individually optimized spinal balance and physiologic posture. Thus, preoperative planning and preparation is crucial for achieving this goal [6,7]. However, PSO is a technically demanding procedure and has been associated with increased blood loss, new postoperative motor deficits, pseudarthrosis, implant failure, and the loss of correction [3,[8], [9], [10]]. Increased blood loss may further obscure the situs overview and prevent the achievement of optimal spinal realignment. Thus, the established lordosis restoration goals can be easily missed and, ultimately, result in unfavorable postoperative spinopelvic parameters and continued pain and disability, which may necessitate even more challenging revision surgery [11,12].

Patient-specific instruments (PSI) with three-dimensional (3D)-printed guides have been introduced for several challenging orthopedic procedures [13,14]. The guides are designed on the basis of the patient's computed tomography (CT) scan. PSI has been established for complex corrective osteotomies of the lower leg and optimal prosthesis alignment [15,16]. Currently, PSI is also more routinely applied in the correction of malunions of the upper and lower extremities and in margin-respecting orthopedic tumor surgery [15,[17], [18], [19]]. Moreover, PSI has been found to be particularly valuable for pedicle screw insertion during spine surgery, promoting more accurate screw placement compared with the free-hand (FH) technique [20,21]. Recent reports have presented the first cases treated with PSI-guided vertebral resection osteotomies and extended PSOs for patients with severe kyphoscoliosis secondary to ankylosing spondylitis [22,23]. However, in addition to these few cases, PSI has not been employed in a standardized manner or compared with the conventional FH technique in terms of any corrective spinal osteotomies, including PSO.

This study, therefore, aimed to investigate the feasibility and accuracy of preoperatively planned lordosis gain during PSI-guided PSOs and compare it with the conventional FH technique. The secondary objective was to compare the congruence of posterior column elements after osteotomy closure between these two techniques.

## Methods

### Study characteristics

After obtaining an approval from the local ethics committee (BASEC-Nr. 2020-01326), six fresh frozen thoracolumbar spine cadavers (T10–Sacrum) from human donors (Science Care, Phoenix, AZ, USA) were used for this study. CT scans of all specimens were obtained before dissection. The cadavers were stored at −20°C until dissection. After the specimens were carefully dissected, the paraspinal musculature was removed to reveal the spinous process and laminae of each vertebra. Each specimen underwent three PSOs at the L1, L3, and L5 levels, leading to 18 (3 × 6) PSOs in total. Nine PSOs were performed using the conventional FH technique and nine using the PSI. To minimize selection bias through inter- and intracadaveric changes, these two techniques were alternatively assigned to the randomly ordered specimen. As such, each specimen underwent either two FH-PSOs (L1 and L5) and one PSI-guided PSO (L3) or vice versa, with each technique being used three times at each vertebral level (L1, L3, and L5).

### PSI planning and guide development

**Fig. 1:**
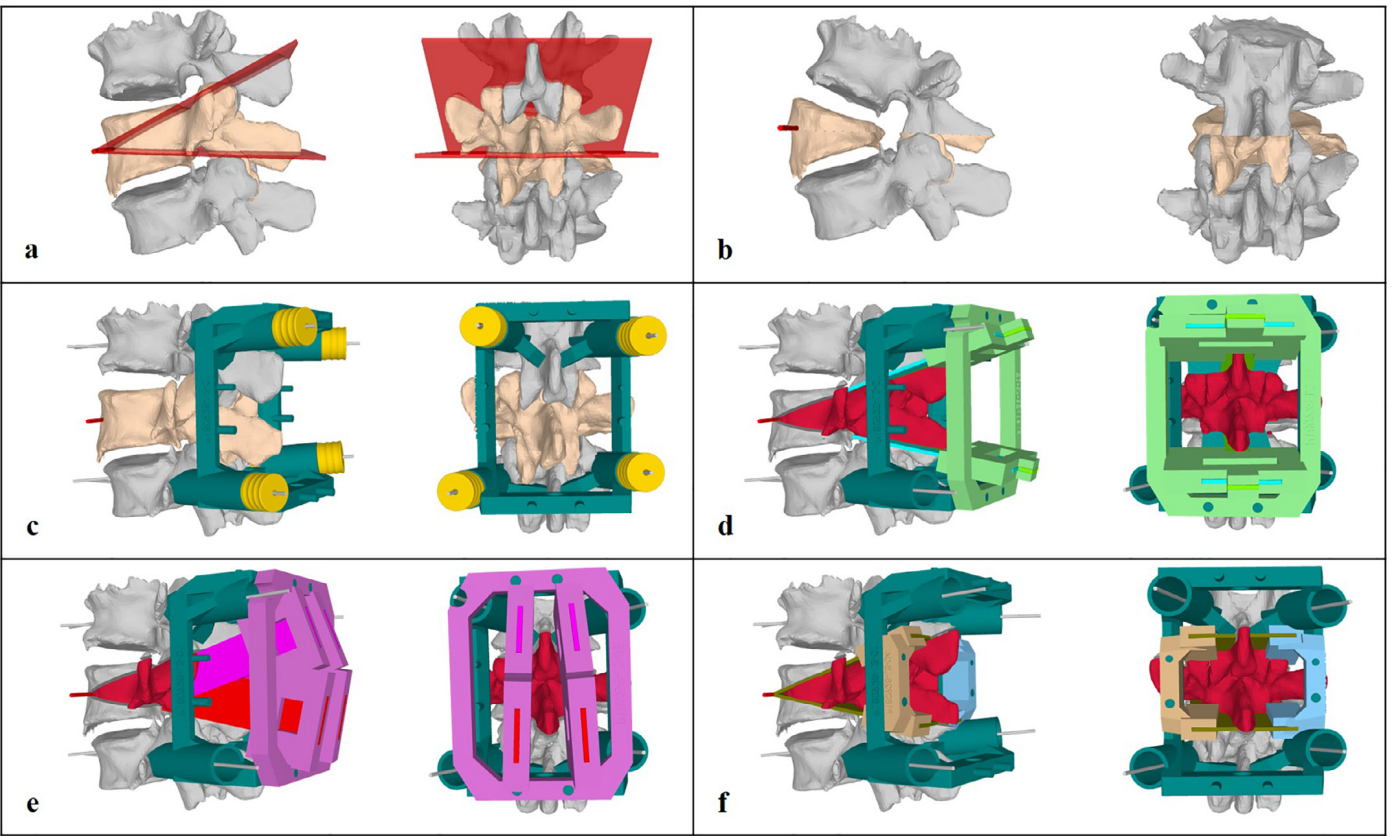
**Patient-specific instrument planning.** A) Cranial and caudal osteotomy planes. B) Simulation of alignment after pedicle subtraction osteotomy (PSO) wedge closing. Note that the inferior aspect of the lamina of the PSO vertebra was left intact to achieve a bony bridge posteriorly. C) Ground block (dark green) attached to the adjacent upper and lower vertebrae and fixated with 2.7-mm wires through yellow drill guides at first. The drill guides are later removed, and cannulated pedicle screws are inserted (not shown) to fix the ground block. D) Horizontal osteotomy guide (light green) attached to the ground block. E) Vertical osteotomy guide (purple) attached to the ground block. F) left (brown) and right (light blue) horizontal osteotomy guides for anterior vertebral body osteotomies.

Using the obtained CT data, triangular surface models of all vertebrae were generated. A computer-aided design surgical planning software CASPA (Balgrist CARD, Zurich, Switzerland) was then used to plan the PSO wedge, with a cranial and caudal osteotomy plane meeting each other approximately 0.5 cm posterior to the anterior border of the vertebral body in a 30° angle (Fig. 1A). The cranial osteotomy plane cut the posterior structures (i.e., spinous process, laminae, and inferior articular process) of the upper adjacent vertebrae and entered the vertebral body of the PSO at the cranial border of the pedicle. The caudal osteotomy plane cut the spinous process and lamina of the PSO vertebra and entered the PSO vertebra body caudal to its pedicle. During all PSOs, some lamina and both inferior articular processes of the PSO vertebral body remained intact, allowing bony contact between the posterior structures after the wedge closure (Fig. 1B).

A PSI design comprising the following five parts was developed: the ground block, two posterior osteotomy guides, and two anterior osteotomy guides (left and right). The ground block was constructed with contact areas at the laminae and around the pedicle screw insertion sites of the cranially and caudally adjacent vertebrae (Fig. 1C). The osteotomy guides were mountable on the ground block. The first posterior osteotomy guide comprised the gauges that horizontally cut the spinous process, laminae, and facet joints of the PSO vertebra as well as the cranial adjacent vertebra (Fig. 1D). The second posterior osteotomy guide comprised the posterior vertical osteotomy gauges that allowed en-bloc removal of a quadrangular osteotomy piece, including the osteotomized lamina and spinous process (Fig. 1E). The two anterior osteotomy guides (left and right) were planned for the wedge osteotomy of the vertebral body (Fig. 1F).

**Fig. 2:**
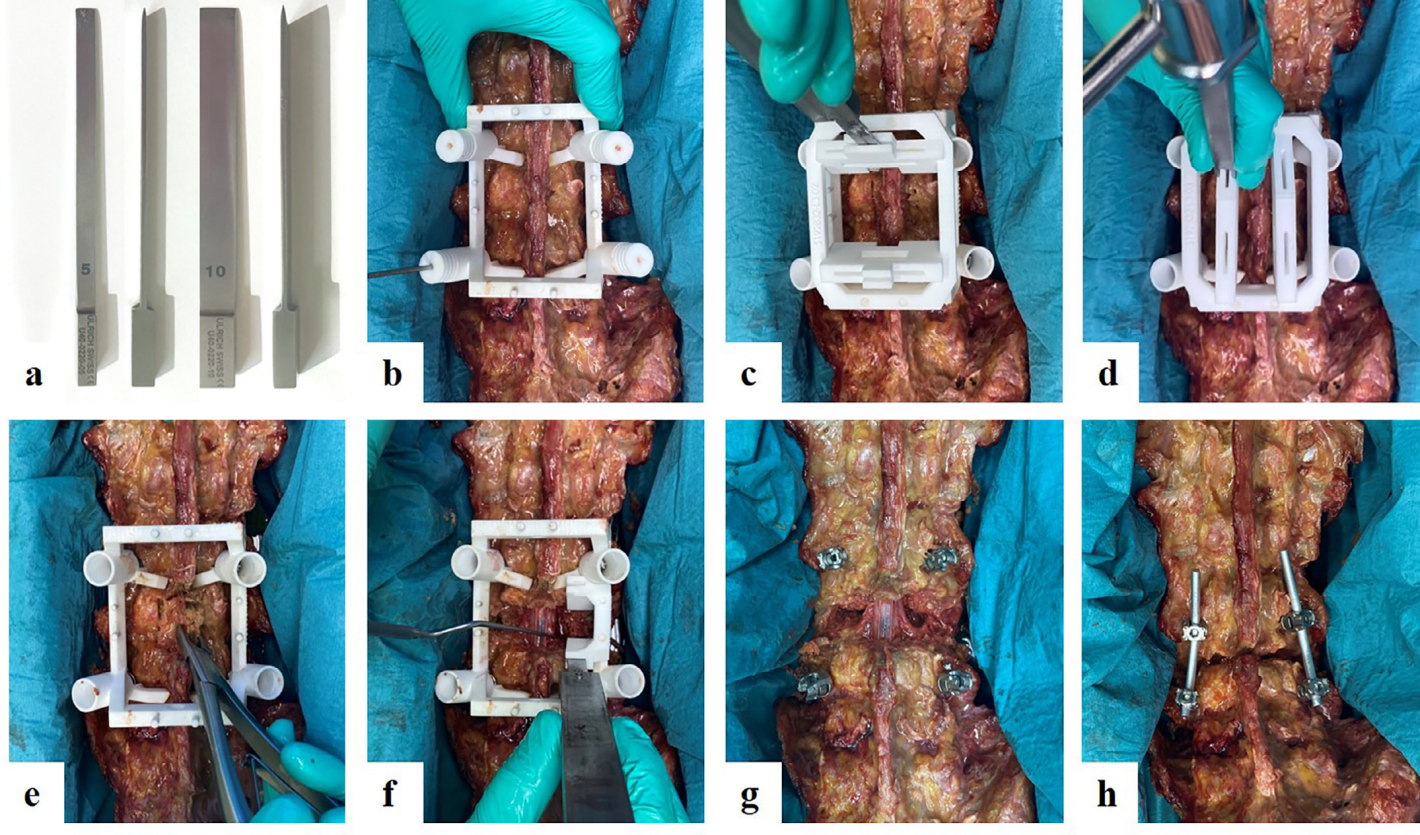
**Surgical technique of a patient-specific instrument-guided pedicle subtraction osteotomy (PSO).** A) Front and side view of 5-mm and 10-mm depth-limited osteotomes with a stopping element 80-mm distant to the tip end. B) An example of a PSO of vertebra L3 is shown. The ground block is placed over L2 to L4. A 2.7-mm drill bit (L4 on the left) is used to predrill the trajectory of the pedicle screws, which are later inserted to fix the position of the ground block. C) Mounted posterior horizontal osteotomy guide with an inserted chisel. D) Mounted posterior vertical osteotomy guide. E) En-bloc removal of the osteotomized posterior structures revealing the spinal canal. F) After bilateral removal of the L3 pedicles and L2/3 facet joints, 30° wedge osteotomy of the vertebral body is performed. The right sided anterior osteotomy is illustrated, which is later followed by the left anterior osteotomy. The nerve roots were protected with a retractor. G) After completing the osteotomy, the osteotomy guides are removed. H) The PSO is closed and fixed with vertical rods.

For the PSI group, special osteotomes with a width of 5 and 10 mm were constructed using a depth-limiting stopper after a cutting length of 80 mm (Ulrich AG, St. Gallen, Switzerland) (Fig. 2A).

The length and width of the cutting gauges were adapted to each vertebra to ensure that the depth-limited osteotomes did not penetrate the spinal canal and nerve roots during posterior osteotomy or the anterior cortex of the vertebral body during anterior osteotomy.

### PSO surgical technique

All PSOs were performed by an experienced spine surgeon. The goal of each PSO was to gain a 30° lordosis with both the FH and PSI techniques. In the FH group, the spine surgeon performed PSO as he would on living patients, except for the special goal of achieving the 30° of lordosis correction.

After defining the level of the PSO, the ground block was set and fixed using four pedicle screws (cannulated poly-axial pedicle screws; Medacta International, Castel San Pietro, Switzerland). The ground block contained four removable, cannulated cylinders that served as drill guides (2.7 mm) before pedicle screw placement above and below the index level. Thereafter, cranial and caudal horizontal posterior osteotomies were performed, followed by vertical posterior osteotomies (Fig. 2B and C). The underlying spinal canal and nerve structures were exposed by removing the osteotomized bone (Fig. 2D). The anterior osteotomy was then performed using the respective mounted anterior osteotomy guides (Fig. 2E). The dural sac and nerve roots were retracted to provide the chisel access to the vertebral body. The anterior osteotomy guide predefined the osteotomy of the lateral and anterior aspects of the vertebral body. The posterior and central aspect of the vertebral body, which lies directly anterior to the spinal canal, had to be removed manually using curved rongeurs. After the removal of the bony wedge created in the vertebral body, the osteotomy was closed and fixed with a screw–rod construct (Fig. 2F).

### PSO evaluation

**Fig. 3:**
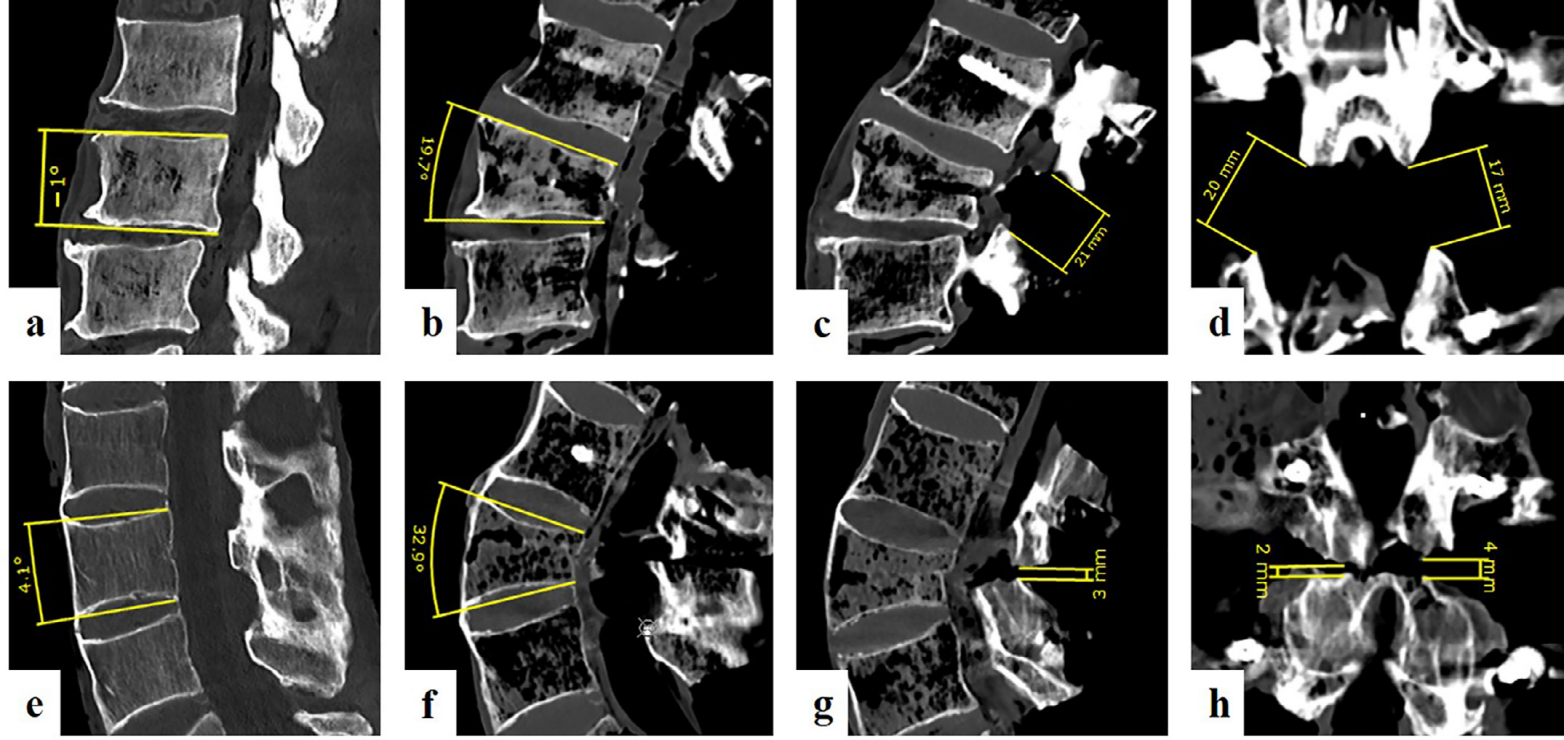
**Lordosis correction and osseous gap, two illustrative cases.** A–D: Example of a free-hand pedicle subtraction osteotomy (PSO). Vertebral lordosis angle corrected from 1° of kyphosis to 20° of lordosis = 21° of lordosis gain (A and B). Large posterior osseous gaps encountered on paramedian sagittal and coronal computed tomography images (C and D). E–H: Example of patient-specific instruments (PSI)-guided PSO. The correction of 4° of lordosis to 33° of lordosis = 29° of lordosis gain (E and F). Small posterior osseous gaps encountered with the PSI technique.

The time required to execute PSO was measured in both groups, starting from the identification of the PSO level until closing and fixation. After each PSO, CT images were obtained to measure the accuracy of the planned versus executed PSO. CT measurements were performed while being blinded to the technique used. The lordosis gain within the PSO level was calculated as the difference between pre- and postoperative sagittal angulation between the upper and lower endplate of the PSO vertebra (Fig. 3 A-B; E-F). Furthermore, the osseous gaps between the posterior elements after osteotomy reduction were measured through CT scan (Fig. 3C-D; G-H). In both the coronal and sagittal planes, the shortest distances between the dorsal elements were measured on the left and right, with the average of the two values being recorded. The sagittal and coronal gaps as well as the average of the sagittal and coronal values were separately compared between both groups.

**Fig. 4:**
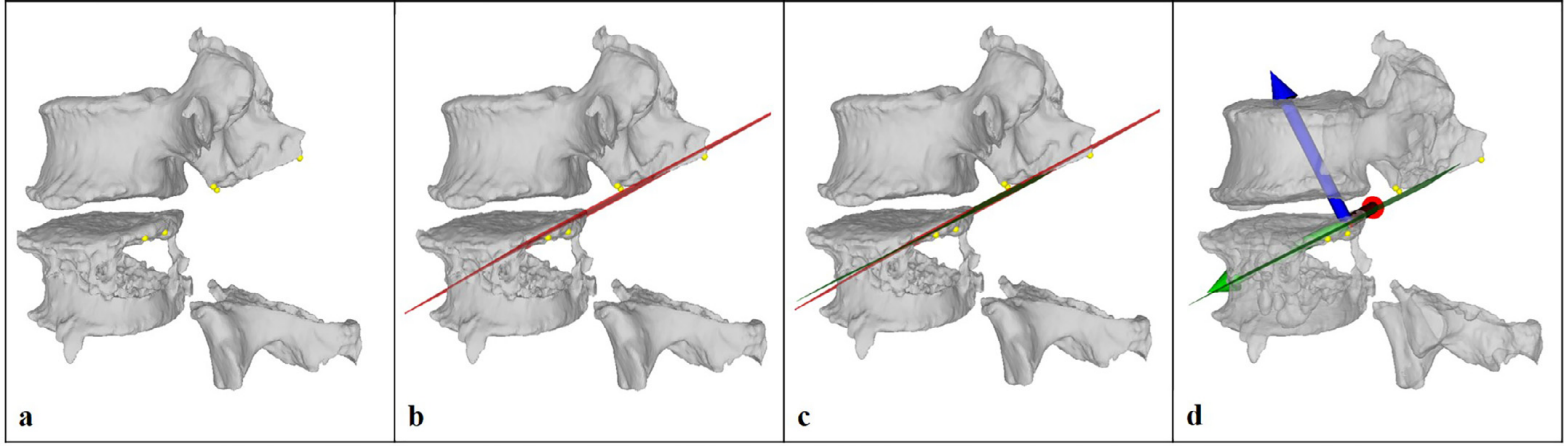
**Accuracy of executed versus planned patient-specific instrument-guided PSO.** A) Yellow marker points on cranial lamina and vertebral body of cranial osteotomy plane. B) Red plane = calculated best fitting reconstructed osteotomy plane according to the yellow marker points. C) Angle measurement between preoperatively planned (green) and executed (red) plane. D) Perpendicular distance of the yellow marker points to the preoperatively planned osteotomy plane was measured (in the direction of the blue arrow).

In the PSI group, the accuracy of the planned versus executed upper and lower osteotomy planes of the PSO was separately evaluated. For this purpose, separate CT scans were performed and 3D-reconstructed after reopening the PSO wedge for better imaging of the osteotomy planes. In total, 6–8 marker points were set at the cranial and caudal osteotomy planes (Fig. 4a), half of which were set at the surface of the osteotomized laminae and the other half at the osteotomy entry of the vertebral body. The executed osteotomy plane was 3D-reconstructed by calculating the best fit of the marker points on the surface of the vertebral osteotomy (Fig. 4B). The largest possible angle between the planned and executed osteotomies was recorded (Fig. 4B). In addition, the perpendicular distance between each of these markers to the planned osteotomy plane was measured, with the overall average and average of the anterior and posterior marker points being recorded (Fig. 4D).

### Statistical analysis

Statistical analyses were performed using SPSS (version 25; IBM, Armonk, NY). Data were nonnormally distributed using Shapiro–Wilk tests. Intergroup comparisons were performed using the Mann–Whitney U test. The level of statistical significance was set at p < 0.05 (for two-tailed analyses).

## Results

In total, 18 PSOs were performed, 9 with the PSI and 9 with the FH technique, on six human thoracolumbar cadavers. Median age of the donors—five males and one female—was 65 (range 54–73) years. None of the cadavers revealed any spinal alignment pathology or had previously undergone any surgical intervention on the thoracolumbar spine. No complications such as neural damage or unintentional fractures were observed during the PSO procedures in either group. Median time required to execute PSO was 18:22 (range 10:22–26:38) min and 14:14 (range 10:13–22:16) min in the PSI and FH groups, respectively, with no significant difference between the groups (p = 0.489) (Table 1).

**Table 1:** PSI-guided PSO versus FH-PSO

	PSI (n = 9)		FH (n = 9)		p-Value
	Median	Range	Median	Range	
Time (min:s)	18:22	10:22–26:38	14:14	10:13–22:16	0.489
Preoperative vertebral lordosis [°]	−4	−11–(+10.0)	−2	−11–(+5)	0.825
Postoperative vertebral lordosis [°]	−32	−38–(−16)	−26	−35–(−10)	0.102
Gain of lordosis [°]	**29**	23–31	**21**	13–34	**0.015**
Difference to 30° aim [°]	**1**	0–7	**9**	4–17	**0.003**
Osseous gap sagittal [mm]	**5**	0–9	**10**	3–26	**0.043**
Osseous gap coronal [mm]	**4**	0–9	**11**	3–28	**0.027**
Average osseous gap ($\frac{sagittal+coronal}{2}$) [mm]	**5**	0–9	**11**	3–27	**0.043**

PSO, pedicle subtraction osteotomy; PSI, patient-specific instruments; FH, free-hand. Negative values (−) of lordosis represent actual lordosis, whereas positive values (+) indicate kyphosis. Bold-faced values indicate statistical significance.

The PSI group had a significantly higher lordosis gain than the FH group (29°, range 23–31° vs. 21°, range 13–34°), respectively (p = 0.015). The PSI-guided PSO was significantly more precise and accurate than the FH-PSO, with a median deviation from the targeted 30° correction of 1° (range 0–7°) versus 9° (range 4–17°), respectively (p = 0.003). Moreover, the PSI group exhibited a significantly smaller residual osseous gap between the posterior parts of the closing wedges (i.e., laminae) compared with the FH group (p = 0.043), with a median overall osseous gap of 5 (range 0–9) mm and 11 (range 3–27) mm in the PSI and FH groups, respectively (Table 1; Fig. 3).

Assessment of the planned versus executed osteotomy in the PSI group revealed an angular difference of 3° (range 1–12°) with similar findings in both the cranial and caudal osteotomy planes (Table 2). The translational offset of the osteotomy planes at the level of the lamina and vertebral body entry point was 1 (range 0–6) mm and 1 (range 0–4) mm, respectively, with the cranial and caudal osteotomy planes revealing similar values (Table 2).

**Table 2:** Planning versus execution of PSO with PSI

	Median	Range
Overall deviation angle [°]	3	1–12
Cranial osteotomy plane [°]	3	1–6
Caudal osteotomy plane [°]	3	1–12
Overall posterior translational distance [mm]	1	0–6
Cranial osteotomy plane [mm]	1	0–3
Caudal osteotomy plane [mm]	1	0–6
Overall vertebral body translational distance [mm]	1	0–4
Cranial osteotomy plane [mm]	1	1–3
Caudal osteotomy plane [mm]	1	0–4

PSO, pedicle subtraction osteotomy; PSI, patient-specific instruments. Overall deviation angle and overall translational distance = median and range of both the cranial and caudal osteotomy planes.

## Discussion

The present study aimed to investigate the value of PSI in the planning and execution of PSOs. Our results revealed that PSI-guided PSO was safe and did not cause any intraoperative complications in the cadaver model. A comparison between the FH- and PSI-guided PSOs showed that the latter promoted more extensive lordosis gain, achieving the set gain of 30°, and had greater precision and accuracy than the standard technique. The PSI technique allowed for achieving not only the closure of the wedge in the vertebral body but also bone-on-bone contact of the posterior elements (i.e., laminae of PSO vertebra and upper adjacent vertebra).

The time to perform a PSO was similar, but slightly and non-significantly increased with the PSI-guided technique (18 min 22 sec), compared to freehand (14 min 14 sec). However, performance of a new operative technique such as PSI may involve an effort-intense learning curve, before it may be compared to a more routine procedure, which the investigating surgeon is more familiar with. With continuation of this technique and further optimization of the PSI design and surgical tools (i.e. depth-limited osteotomes), a further increase in surgical performance is plausible.

The PSI design and osteotomy technique in our study was inspired by the original PSO reports by Thomasen as well as further technique descriptions by Bridwell et al. [2,4,5] In their illustrative descriptions, the posteroinferior lamina and inferior articular process of the PSO vertebra was left intact, which resulted in a continuous posterolateral bony bridge. This technique stands in contrast to that published by other authors who have described the complete removal of the lamina and facet joints of the PSO vertebra. [1,24,25] The complete removal of the posterior structures was also the approach used by the investigating spine surgeon in this study in the FH-PSO group. This may explain the significantly larger osseous gap between the posterolateral structures in the FH group on sagittal and coronal CT slices. However, using the technique could preserve the facet joint caudal to the PSO, which should be surgically addressed to promote bony fusion at this site in cases with unossified facet joints.

In 2018, Pijpker et al. [22] reported the first case to receive a PSI-guided complex coronal and sagittal spinal correction osteotomy. After performing a bone–disc–bone T11/12-extended PSO on a child with congenital kyphoscoliosis, they reported an astonishing clinical and radiographic postoperative course. Contrary to our approach, they constructed separate guides for the cranial and caudal osteotomies. However, these guides were used for orientation only and were removed after the initial osteotomy, which was then completed without the guide. Tu et al. [23] further reported a case series of nine patients who underwent spinal osteotomy realignment procedures using PSI for patients with severe ankylosing spondylitis; they used PSI for screw placement and as a reference for the osteotomy. However, although their titanium templates seemed to provide significant aid for wedge osteotomy orientation, they did not provide closed guidance with cutting gauges.

In contrast to these previously reported PSI designs, the approach described herein allowed for a more sophisticated PSI technique that not only orientated the osteotomy trajectories through the posterior elements but also separately guided the wedge osteotomy of the vertebral body. This study is also the first to compare the PSI approach to the conventional FH technique. Our findings showed that the PSI technique required, on average, more time than the FH technique, although no significant differences were observed. We believe that these differences could be attributed to 1) the lack of experience with PSI in complex spine surgery and 2) the extra-manufactured depth-limited osteotomes. The depth-limited osteotomes were the major concern of the spine surgeon, given that the shape of these osteotomes required more time for osseous penetration and removal than the standard osteotomes used in the FH group. Although the concept of the PSI and depth-limited osteotomes showed superior results in our study, the described design of the instruments still leaves room for improvement in future applications.

### Clinical significance

In general, optimal sagittal realignment requires a horizontal gaze and head position over the pelvis, creating an ergonomically efficient standing and walking alignment. [26] On the basis of the formula (lumbar lordosis [LL] = pelvic incidence [PI] ± 9°), spinopelvic realignment procedures have generally aimed to achieve an LL that equals the PI, with the C7 vertebra being centered over the sacrum (sagittal vertical axis < 5 cm) [11,27]. However, age- and individual-specific differences need to be additionally considered [28,29]. Failure to achieve these goals have been associated with poor patient outcomes and increased reoperation rates [30], [31], [32]. Moreover, failure to restore an appropriate LL leads to increased force loadings at adjacent segments, possibly triggering adjacent segment disease, proximal junctional kyphosis, and implant failure [33], [34], [35], [36], [37]. The incongruence between surgical planning and execution (i.e., 1° with the PSI technique vs. 9° with the FH technique) might have considerably impacted patient outcomes, accounting for the difference between surgical success and failure.

The smaller posterolateral osseous gaps may possibly promote faster bony fusion, which, in turn, could facilitate less pseudarthrosis and less hardware failure. Accordingly, studies have reported rod fracture rates of up to 22% within the first 2 years following PSO [10]. Although rod material, thickness, and contour angulation have been identified as risk factors for rod fracture, the association between posterior osseous gap and rod fracture has, to our knowledge, not yet been investigated. [10,38] The advantage of a small osseous gap must, however, be weighed against the possible disadvantages such as less overview during wedge closure and possible neural compression by the remaining posterior structures. To avoid such complications, Bridwell suggests enlarging the spinal canal similar to that during midline decompression to inspect the dura and spinal canal after osteotomy closure [4].

Although the aforementioned studies have led to the optimization of preoperative planning and postoperative realignment control, intraoperative control of the amount of correction remains challenging. Although the criteria for optimal spinal alignment have been well defined, the achieved correction still strongly relies on the surgeon's subjective intraoperative decisions. In cases of complex spinal osteotomy with vigorous bleeding and at-risk neural structures, spinal surgeons' capabilities to simultaneously ensure that the preoperative alignment goals are achieved may be limited. Although Blondel et al. [39] suggested intraoperatively obtaining long-cassette radiographs to control the execution of the preoperative plan, the efficacy and practicability of this approach has, to our knowledge, not been investigated yet. We believe that the intraoperative lordosis measurements, reopening of a closed wedge, and extending the osteotomy in case of insufficient deformity correction are time-consuming and not routinely practicable in a surgery with massive blood loss, such as PSO. PSI can be a robust approach for shifting intraoperative decisions to preoperative planning and preparation.

The herein described PSI-guided PSO approach further offers the possibility of individual adaption of the wedge angle to the patient's anatomy. Although the wedge angle of 30° was selected for simplicity and comparability between the PSI and FH techniques, this can be modified in the approximate range of 15–35°. Furthermore, the wedge could be rotated in the craniocaudal and anteroposterior axes to perform asymmetric PSOs. By doing so, deformities in both the sagittal and coronal planes could be corrected, for example, in thoracolumbar kyphoscoliosis [40]. However, this falls outside the purview of this pilot study but provides an outlook for future work.

### Limitations

The potentially most prominent limitation of this cadaveric pilot study is that the spinal specimens investigated were in the same position during CT and the osteotomy procedures. This eliminates potential issues with positional changes in lordosis. The fit of osteotomy guides set over three vertebrae with flexible lumbar discs in between may be limited in humans, considering that CT images are obtained in the supine position and surgery is performed in prone position. Therefore, the performance of this system in vivo needs to be evaluated. Another difference between this cadaver study and real intraoperative situations is the lack of blood, scar tissue, and intraspinal fluid, which can lead to an unrealistically good overview during the procedure. Further in vivo evaluation might reveal additional limitations in such conditions, which are difficult to simulate in a cadaver. Furthermore, the accuracy measurement of the planned versus executed osteotomies illustrated in Fig. 4 is yet to become a standardized approach. However, we believe this to be a reliable technique that could also be applied in other osteotomy accuracy measurements.

## Conclusion

The present study showed that PSI-guided PSO is feasible and more accurate in achieving the planned lordosis angle compared with the traditional FH technique in a cadaver model. Further osseous gaps can be reduced, potentially promoting higher fusion rates in vivo. However, more studies on PSI-guided PSO in real patients are needed to determine its clinical utility.

## Declarations of Competing Interests

The authors declare that they have no known competing financial interests or personal relationships that could have appeared to influence the work reported in this paper.
